# Strategies for Survival of *Staphylococcus aureus* in Host Cells

**DOI:** 10.3390/ijms26020720

**Published:** 2025-01-16

**Authors:** Huiling Xu, Shengnan Wang, Xiaoting Liu, Muzi Li, Xiaozhou Wang, Huahua Chen, Chaonan Qu, Yongxia Liu, Jianzhu Liu

**Affiliations:** 1 College of Veterinary Medicine, Shandong Agricultural University, Tai’an 271018, China; huilingxu0319@163.com (H.X.); wangshengnan8989@163.com (S.W.); sdlxt25@126.com (X.L.); sdau_wxz@163.com (X.W.); qcn264200@163.com (C.Q.); 2 Shandong Provincial Key Laboratory of Zoonoses, Shandong Agricultural University, Tai’an 271018, China; limz395430@163.com (M.L.); 19860939156@163.com (H.C.)

**Keywords:** *Staphylococcus aureus*, internalization, toxin, EVs, biofilms, SCVs

## Abstract

*Staphylococcus aureus*, a common pathogen, is capable of producing a significant array of toxins and can develop biofilms or small colony variants (SCVs) to evade detection by the immune system and resist the effects of antibiotics. Its ability to persist for extended periods within host cells has led to increased research interest. This review examines the process of internalization of *S. aureus*, highlighting the impact of its toxins and adhesion factors on host cells. It elucidates the intricate interactions between them and the host cellular environment, thereby offering potential strategies for the treatment and prevention of *S. aureus* infections.

## 1. Introduction

*Staphylococcus aureus* (*S. aureus*), a facultative intracellular microorganism, demonstrates the ability to survive within host cells and is responsible for a variety of diseases in both humans and animals. These diseases encompass pyogenic inflammation and sepsis, among others. The emergence of some antibiotic resistant strains of *S. aureus* poses a serious threat to global public health [1]. *S. aureus* can produce diverse toxins, immune evasion factors, and protein and non-protein factors, playing a role in its adhesion, invasion, and immune avoidance [2]. The formation of *S. aureus* biofilms and small colony variants (SCVs) also contribute to the persistence of bacterial infection [3].

The essential aspect of the life cycle of *S. aureus* is its capacity to invade host cells, a process predominantly initiated by the bacterium. Several studies have clarified the significant roles played by virulence factors and adhesion factors in the interactions between *S. aureus* and its host, facilitating the bacterium’s adept evasion of the host’s immune defenses. *S. aureus* binds to the surfaces of cells or tissues through surface adhesion proteins [4], triggers Rho GTPase, induces rearrangements in the cytoskeleton, and subsequently invades host cells through a mechanism resembling a zipper [3].

Current studies are centered on the enduring role of *S. aureus* in cellular longevity. There is an increasing body of evidence indicating that *S. aureus* employs various strategies to evade detection and proliferate within host cells. To enhance its survival within these cells, *S. aureus* minimizes the release of toxins, thereby reducing its visibility to the immune system [5]. Furthermore, research has demonstrated that low-virulence strains of *S. aureus* are more frequently associated with the occurrence of bacteremia compared to their highly virulent counterparts [6]. *S. aureus* can also be converted into biofilm or SCVs, evade antibiotic killing, and adapt to different harsh environments to survive. There is an intricate mechanism of how *S. aureus* is internalized into cells, what factors influence its internalization, what role its toxins play, and how it escapes from cells. This review will summarize the internalization of *S. aureus*, their complex relationship with their hosts, and their ultimate fate.

## 2. Internalization of *S. aureus*

**Figure 1:**
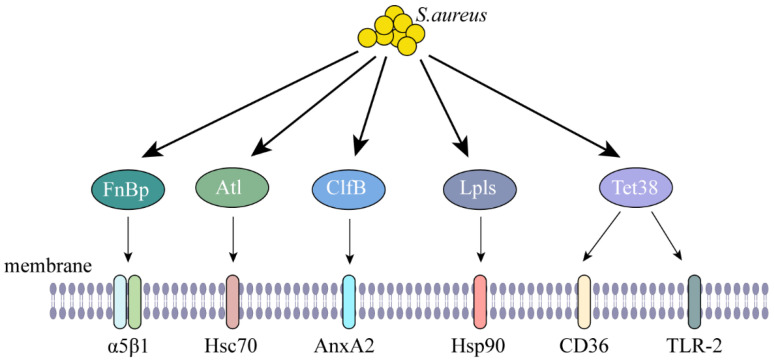
**Upon internalization, the adhesion factors of *S. aureus* act on cell surface receptors.** FnBp binds to α5β1 on the cell membrane. Atl binds to Hsc70 on the cell membrane. ClfB binds to AnxA2 on the cell membrane. Lpls binds to Hsp90 on the cell membrane. Tet38 binds to CD36 and TLR2 on the cell membrane.

Internalization of *S. aureus* is mediated by adhesion factors on its surface, such as fibronectin (FnBP), fibrinogen (FgBP), collagen-binding (Cna) proteins, and clumping factors (ClfA and ClfB), which bind to cell surface fibronectin receptors and promote bacterial internalization [7]. There are many studies that have shown that some adhesion factors of *S. aureus* attach to cell surface receptors, facilitating the internalization of *S. aureus*. Among these factors, FnBp engages with the cellular integrin α5β1, facilitating the invasion of *S. aureus* into respiratory epithelial cells [8,9]. In certain strains of *Staphylococcus epidermidis*, as well as in *staphylococci* that do not possess the homologous protein of FnBP, an alternative mechanism for bacterial internalization has been identified. This mechanism involves the *staphylococcal* autolysin (Atl), which interacts with the heat-shock cognate protein Hsc70 found on endothelial cells, thereby facilitating the entry of bacteria [10]. The interaction between the membrane-associated protein A2 (AnxA2) and ClfB in bovine mammary epithelial cells was evidenced through co-immunoprecipitation (CO-IP) experiments. Notably, treatment with purified ClfB resulted in an enhanced internalization of *S. aureus*, while a knockdown of AnxA2 led to a reduction in *S. aureus* levels. Furthermore, the disruption of actin polymerization was observed, indicating that ClfB plays a crucial role in the invasion process, particularly in comparison to bacterial adhesion [11]. Lipoprotein-like lipoproteins (Lpls) are identified as significant contributors to the internalization of *S. aureus* strain USA300. This process is facilitated by the binding of Lpls to Hsp90, which promotes actin polymerization, enabling the entry of *S. aureus* into human keratinocytes [12]. The tetracycline resistant protein Tet38 from *S. aureus* exhibits a specific binding affinity for the cell membrane surface receptors CD36 and toll like receptor 2 (TLR2). This interaction plays an important role in the entry of *S. aureus* into human lung adenocarcinoma cells [13] (Figure 1, Table 1). We employed AlphaFold 3 to predict the interactions between adhesion factors and cell surface receptors. Our findings indicate that, with the exception of Atl-Hsc70, Tet38-TLR2, and Tet38-CD36—where the iPTM + PTM values did not exceed 0.75—all other assessed interactions exhibited iPTM + PTM values greater than 0.75, implying the likelihood of these interactions (Table S1). Notably, interaction sites were identified in Atl-Hsc70 and Tet38-CD36; however, no interaction was observed between Tet38-TLR2, despite prior reports suggesting such an interaction (Figure S1).

Altering cellular conditions can also affect the internalization of *S. aureus*. Studies have demonstrated that *S. aureus* can promote its uptake by recruiting actin, and that the application of the actin polymerization inhibitor Cytochalasin D has been shown to diminish the uptake of *S. aureus*, while a temporary enrichment of actin is noted at the sites of bacterial adhesion [29], Additionally, *S. aureus* is capable of enlisting clathrin to aid in its entry into host cell [30]. When knockdown of filaggin occurs, it promotes the internalization of *S. aureus* into keratinocytes [31]. Investigations have revealed that cytokines can influence the internalization of *S. aureus* within cells. When interferon-γ (IFN-γ), a cytokine associated with T helper 1 (Th1) responses, was administered prior to the exposure of cells to *S. aureus*, a significant reduction in the internalization of the bacterium was noted. Similarly, the addition of interleukin-10 (IL-10) or interleukin-17A (IL-17A) resulted in lower levels of internalization compared to the control group. However, these differences did not reach statistical significance. Additionally, pretreatment with interleukin-4 (IL-4) or interleukin-13 (IL-13) did not result in any observable changes in the levels of internalization [31]. Proteinase K, an endoprotease, was used to inhibit the expression of fibronectin in *S. aureus*, leading to a decrease in the quantity of internalized cells [31]. The internalization of *S. aureus* allows it to evade the action of certain antibiotics that are unable to penetrate the cellular membrane, presenting a significant challenge for prevention, control, and treatment strategies.

## 3. Survival Strategies for *S. aureus*

The general prevalence of *S. aureus* is due to its virulence factors and genomic plasticity, which either produce strong toxins that cause transient infections or, in the form of colonies, form biofilms, causing long-term infections [32]. How do these toxins work to kill host cells and carry out cell-to-cell invasion?

### 3.1. Alpha-Hemolysin (α-Toxin)

**Figure 2:**
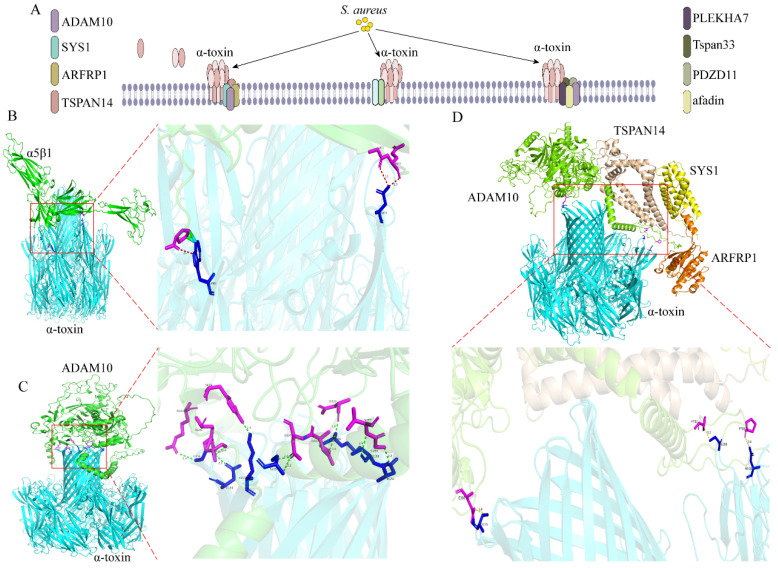
**Interaction between α-toxin and host cells.** (**A**) The α-toxin punches holes in the cell membrane in the form of heptameric and allows *S. aureus* to invade the cell by interacting with the host’s ADAM10 or α5β1. SYS1, ARFRP1, TSPAN14, SYS1, ARFRP1, TSPAN14 interact with ADAM10 to affect the toxicity of α-toxins in cells. ADAM10 can also bind to Tspan33, PDZD11, PLEKHA7, and afadin to affect the formation of α-toxin pores. (**B**) 3D structure of α-toxin bound to α5β1. Cyan is α-toxins, green is α5β1. (**C**) 3D structure of α-toxin bound to ADAM10. Cyan is α-toxins, green is ADAM10. (**D**) 3D structure of α-toxin bound to ADAM10, SYS1, ARFRP1, TSPAN14, SYS1, ARFRP1, and TSPAN14. Cyan is α-toxins, green is ADAM10, yellow is SYS1, orange is ARFRP1, wheat is TSPAN14. In the figures, the residues of the *S. aureus* toxins are in blue and the residues of the host protein are in magenta.

Studies indicate that alpha hemolysin, also known as α-toxin, is secreted by 95% of *S. aureus* [33]. The α-toxin is mainly regulated by the positive regulators Agr, sae, and sarA and the negative regulator SigB. The α-toxin is released as a heptameric structure into the β-barrel, traversing the lipid bilayer and subsequently puncturing the target cell membrane to create a hydrophilic transmembrane channel. Additionally, the toxin interacts with the host’s metalloproteinase domain-containing protein 10 (ADAM10), leading to the cleavage of E-cadherin. This action disrupts the actin-based cytoskeleton and facilitates the invasion of the cell [14,15]. The α-toxin can induce the lysis of various cell types, including epithelial cells, endothelial cells, platelets, white blood cells, and certain red blood cells. It has also been shown that α-toxin interacts with α5β1-integrin and induces apoptosis [16]. In addition to ADAM10 and α5β1-integrins, the cellular proteins Sys1 golgi trafficking protein (SYS1), ADP-ribosylation factor 1 (ARFRP1), and tetraspanin-14 (TSPAN14) were identified by modulating ADAM10 to affect α-toxin’s toxicity in cells [17]. Pleckstrin homology domain containing A7 (PLEKHA7) functions as a cytoplasmic auxiliary component of adherens junctions. A notable enrichment of PLEKHA7 has been identified through screening with the α-toxin selection library [18]. Subsequently, ADAM10 was found to bind to tetraspanin 33 (Tspan33), PDZ domain containing 11 (PDZD11), PLEKHA7, and afadin, and they all affected the formation of α-toxin pores [19]. We predicted the interactions between the above proteins by using AlphaFold 3 and found that the iPTM + PTM values were all above 0.75, which is consistent with the experimental results that show that there are interactions (Figure 2, Table 1 and Table S1).

The principal function of α-toxin is to enhance the permeability of cellular membranes or vesicles. It achieves this by facilitating Ca^2+^ influx, which in turn hinders lysosomal acidification and initiates subsequent signal transduction processes. Furthermore, α-toxin plays a crucial role in enabling *S. aureus* to evade the endolysosomal pathway [34,35]. It has been shown that α-toxin promotes the growth and multiplication of other negative bacteria, such as *Pseudomonas aeruginosa*, *Klebsiella pneumoniae*, and *Acinetobacter baumannii*, by preventing acidification of macrophage lysosomes [36]. There is a great deal of controversy about the toxic effects of α-toxin. On the one hand, α-toxin can cause cell death, induce skin barrier destruction and skin inflammation [37], and can also invade the epithelial barrier and destroy lung tissue [38]. Among the α-toxin-induced pulmonary edema, it has been found that α-toxin disrupts the tight junctions of the endothelial cells in the lungs through activation of acidic sphingomyelinase and ceramides, which in turn causes pulmonary edema [39]. Mice infected with mutant strains knocked out of α-toxin caused mild infections, supporting that low-virulence strains will cause chronic infection [40]. On the other hand, studies have shown that there is no correlation between α-toxin severity of infection [41]. It has been reported that α-toxin also affects differentiation of human Th cells. The effect of α-toxin on cell differentiation was assessed by flow cytometry, which showed that the number of deaths of Th1 cells increased with the dose of α-toxins, whereas Th17 cells instead resisted killing by α-toxin. This outcome may be attributed to the higher susceptibility of Th1 cells to Ca^2+^ in comparison to Th17 cells, where an elevation in Ca^2+^ levels could facilitate cell death. Alternatively, it might stem from the differential expression of the receptor ADAM10 between these two cell types. Nevertheless, the extent of ADAM10 interaction with α-toxin appears to exert minimal influence on cell viability, warranting further investigation into the underlying mechanisms [42]. In skin and soft tissue infections caused by *S. aureus*, α-toxin hindered the replication of T cells and dendritic cells and posed a threat to specific T cell responses, and the threat posed by the T cell immune adjuvant CAF01 was rescued by the α-toxin [43]. α-toxin causes great harm to cells and tissues, they have potential value in the discovery of *S. aureus* vaccine. In patients with bacteremia, antibodies against α-toxin have been found to help improve the treatment of bacteremia, and patients with bacteremia who contain few antibodies against α-toxin, resulting in a poor prognosis [44]. For *S. aureus* infections, not only a single α-toxin plays a role, but also other toxins play a non-negligible role, so further research is needed to optimize the vaccine.

### 3.2. Phenol Soluble Modulins

**Figure 3:**
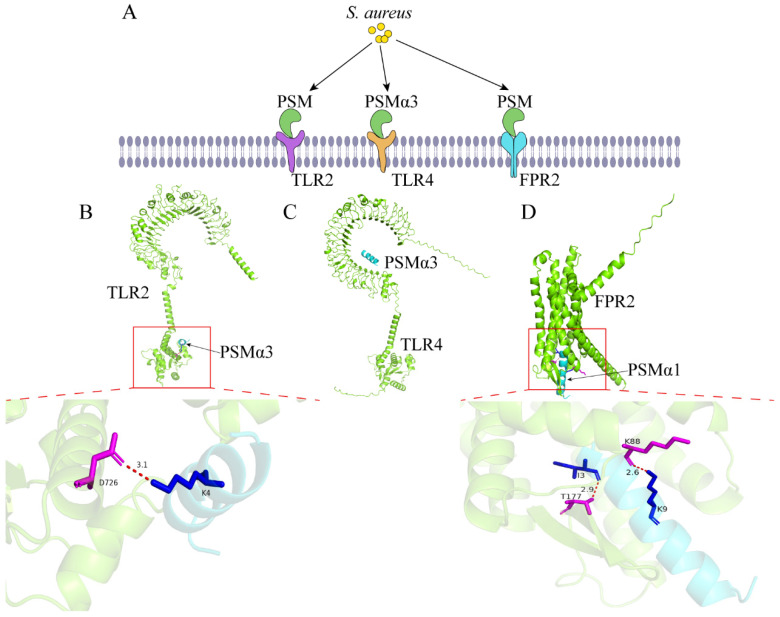
**Interaction between PSM and host cells.** (**A**) PSM interacts with TLR2, TLR4, and FPR2, allowing *S. aureus* to invade cells. (**B**) 3D structure of PSMα3 bound to TLR2. (**C**) 3D structure of PSMα3 bound to TLR4. (**D**) 3D structure of PSMα1 bound to FPR2. Green is host protein, cyan is PSM. In the figures, the residues of the *S. aureus* toxins are in blue and the residues of the host protein are in magenta.

Phenol soluble modulin (PSM) also acts as a virulence factor of *S. aureus* and occupies the same position as α toxin. PSM is mainly categorized into α-types (α1 to α4) and β-types (β1, β2, and δ toxins), which are encoded on mobile genetic elements (MGEs) and governed by the Agr system [45]. PSM plays an important role in assisting bacteria to enter cells, but the presence of lipid bilayers in the cell membrane affects the aggregation of PSM, and kinetic experiments showed that 1,2-dioleoyl-sn-glycero-3-phosphocholine (DOPC) slowed down the aggregation of PSMα1, lipopolysaccharide (LPS) accelerated the aggregation of PSMα1, and DOPC and LPS accelerated the aggregation of PSMα4 [46]. PSMα promotes internalization of *S. aureus* and mediates neutrophil lysis [47]. PSM induces the escape of *S. aureus* from the phagosome to the cytoplasm, and strains lacking PSM are more likely to survive in cells for a long time [48]. It has been shown that PSM does not directly activate TLR2 receptors but rather promotes infection by mobilizing TLR2 ligands in *staphylococci* to interact with TLR2 [49]. However, molecular simulations by 3D modeling have predicted that PSM has the potential to act on TLR4 [20]. It has also been shown that PSMα3 can bind to TLR2 or TLR4 to induce the release of IL-10 and control the production of pro-inflammatory cytokines [22]. We used AlphaFold 3 to predict the interaction of PSM with TLR2 or TLR4, although their iPTM + PTM values were greater than 0.75, and we found that there were interaction sites between PSM and TLR2 and no interaction sites between PSM and TLR4 (Figure 3A–C and Figure S2A, Table 1 and Table S1). PSM acts as a ligand for formyl peptide receptor 2 (FPR2) and induces the release of inflammatory cytophiles IL-8 and IL-1α [21]. Predicting the interaction of PSM with FPR2 using AlphaFold 3 found that both PSMα types bind to FPR2 (Figure 3A,D and Figure S2B, Table 1 and Table S1). PSMα also induces the MyD88 signaling pathway and promotes the production of IL-1α and IL-36α [50,51]. PSMα can induce the expression of a wide array of chemokines and cytokines, thereby promoting the skin’s inflammatory reaction [52]. These studies indicate a crucial function of PSM in immune regulation.

### 3.3. Two-Component Leukotoxins

**Figure 4:**
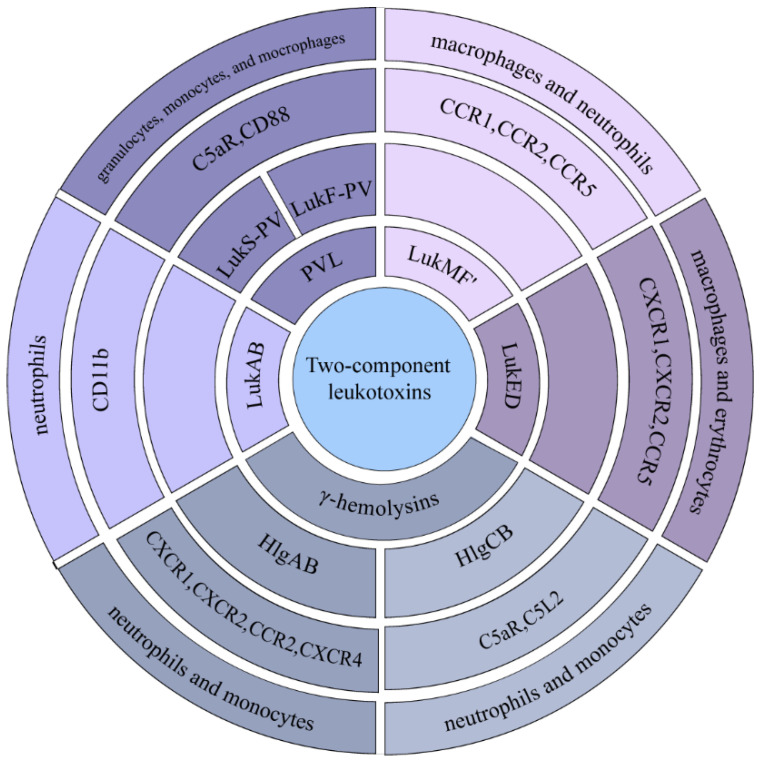
**Two-component leukocidins and the cell surface receptors to which they bind.** HlgAB targets the surface receptors CXCR1, CXCR2, CCR2, and CXCR4 on neutrophils and monocytes. HlgCB targets the surface receptors C5aR and C5L2 on neutrophils and monocytes. LukAB binds the surface receptor CD11b on neutrophils. LukED targets the surface receptors CXCR1, CXCR2 and CCR5 on macrophages and erythrocytes. LukMF’ targets the surface receptors CCR1, CCR2, CCR5 on bovine macrophages and neutrophils. PVL binds to receptors C5aR and CD88 on granulocytes, monocytes, and macrophages.

*S. aureus* is capable of producing several homologous two-component leukocides, which include Panton–Valentine PVL (LukF-PV and LukS-PV), γ-hemolysins (HlgAB and HlgCB), LukED, LukAB, and LukMF’ [53]. These leukocides have two protein subunits, “S” and “F”. They usually bind to the target cell through the “S” subunit and then bind to the “F” subunit, invading the cell as an octamer with four of each subunit, alternating to form an octamer. They act primarily on neutrophils, monocytes, and macrophages, and bind to different surface receptors. PVL binds receptors C5aR and CD88 to promote pore formation [27]. The surface receptors CXCR1, CXCR2, CCR2, and CXCR4 on neutrophils and monocytes are commonly targeted by HlgAB. Following the knockout of the HlgAB toxin, there was a reduction in the bacterial load present within the cells [23]. The other γ-hemolysin, HlgCB, usually targets C5aR and C5L2 and promotes inflammation [23]. LukED acts as a ligand for the surface receptor CCR5, and when interfering with LukED toxicity or mutating the CCR5 gene, bloodstream infections caused by *S. aureus* were significantly reduced [15,24]. LukED can also bind the surface receptors CXCR1 and CXCR2 [25]. LukAB binds the surface receptor CD11b on neutrophils [28]. LukMF’ targets bovine macrophage and neutrophil surface receptors CCR1, CCR2, and CCR5 [26]. The interaction between the components was examined using Alphafold3. The analysis revealed that the iPTM + PTM values for LukED, PVL, and their respective receptors were below 0.75. This does not imply a lack of interaction, as false-negative predictions can occur. Additionally, potential interaction sites between these entities were identified. Consistent with the existing literature, it was noted that the “S” subunit demonstrates a higher affinity for target cells when compared to the “F” subunit (Figure 4 and Figure S3, Table 1 and Table S1).

These pore-forming toxins also have a non-negligible impact on host cells. The pore-forming toxins LukAB and PVL in the Sae regulatory system can induce macrophage death and *S. aureus* escapes [54]. PVL can mediate the escape of *S. aureus* from endosomes and trigger caspase-dependent apoptosis [55]. It has been shown that PVL mainly causes apoptosis through the endogenous pathway of the cell by forming pores on the cell membrane, leading to a reduction in mitochondrial membrane potential and ROS accumulation [56]. PVL is mainly harmful in causing skin and soft tissue infections, and sometimes serious and invasive infections [57,58]. PVL-secreting *S. aureus* has been found in patients infected with SARS-CoV, causing dual infection and promoting necrotizing pneumonia [59]. At present, there exists a necessity to create an efficient toxoid vaccine aimed at neutralizing these two-component leukotoxins.

### 3.4. S. aureus Extracellular Vesicles

There is a class of substances called *S. aureus* extracellular vesicles (EVs), which contain α-toxin, leukocidins, PSM, superantigens, and enzymes. The generation of EVs is affected by many factors, and under low temperatures and hypotonic conditions, the fluidity of the membrane increases, and the efficiency of EVs generation increases [60]. EVs act as a secretory system of *S. aureus* that transports its virulence factors into host cells to help bacteria escape [61]. Research findings suggest that some virulence factors, such as PSMα1-4, promote the production of EVs, and due to their surfactant-like properties can alter the permeability of cell membranes [62]. EVs are crucial in the pathogenesis of *S. aureus* and regulate host cell responses. EVs induce secretion of TNF-α, IL-6, and IFN-γ from mouse splenocytes via TLR2, 4, and 9 receptors [63]. EVs induce epithelial cells to produce pro-inflammatory factors and chemokines, and the formation of LC3-GFP autophagosomes is observed [64]. Research shows that EVs have the potential to cause mitochondrial damage and trigger apoptosis in bovine mammary epithelial cells [65]. So, the impact of EVs is also not negligible, and understanding of their function has also contributed to the understanding of bacterial pathogenesis.

### 3.5. Formation of S. aureus Biofilms

Biofilm is a tissue structure embedded in an extracellular polymer matrix and adhered to the cell surface, and this growth stage is divided into initial attachment, biofilm maturation, and diffusion [66]. *S. aureus* adheres to cellular surfaces by recognizing adhesive matrix molecules known as MSCRAMMs and polysaccharide intercellular adhesins (PIA). Following this initial attachment, the bacteria proliferate and establish microcolonies, which synthesize a variety of polysaccharides, proteins, and extracellular DNA (eDNA). These components collectively contribute to the intricate architecture of the biofilm, facilitating its progression toward maturation. Subsequently, the biofilm experiences a process of diffusion, primarily driven by the secretion of various exogenous enzymes and surfactants that degrade the extracellular polymeric matrix. This degradation allows *S. aureus* to escape from the biofilm and initiate infections in other regions of the host organism [67]. Many substances promote the formation of biofilms, and some studies have found that Mg^2+^ can increase the stiffness of *S. aureus*’s cell walls and encourage biofilm development [33]. PSM also plays a crucial role in the formation of biofilms, which is of great significance for *S. aureus* colonization [68,69]. α-toxin and LukAB are equally synergistic on biofilm formation and also affect phagocytosis by macrophages and cause cell death [70]. It has been discovered that EVs are crucial in the formation of biofilms across various bacteria, and *S. aureus* is no exception; EVs are also able to convert hydrophobic surfaces to hydrophilic surfaces, favoring biofilm formation. Additionally, they diminish the capacity of other bacterial species to establish biofilms [71,72]. Likewise, the development of biofilms primarily accounts for the survival of *S. aureus* in cattle udders [73]. In chronic infections caused by *S. aureus*, the formation of biofilms plays a critical role, as it significantly impedes the effectiveness of antibiotic treatment in eradicating the bacteria.

### 3.6. Small Colony Variants (SCVs)

As a common phenotypic shift in *S. aureus*, small colony variants (SCVs) are crucial in the infection phase of *S. aureus*. In a number of harsh environments, including extreme pH, nutritional deficiencies, stress, antibiotic treatment, etc., a slow-growing bacterial subgroup is formed [74]. SCVs have some specific characteristics, such as small colonies, reduced pigmentation, decreased hemolysin production, up-regulated expression of adhesion genes, increased drug resistance, and slow growth [75]. The downregulation of the Agr quorum sensing system can promote the formation of SCVs. SigB has the potential to diminish virulence and facilitate the biogenesis of SCVs [76,77]. It was shown that the expression of α-toxins and PMSα was reduced in SCVs [78]. It has been shown that when cells are infected with the highly virulent *S. aureus*, the host initiates a degradation pathway, resulting in a decrease in the amount of bacteria in the cell, whereas infecting the cells with a small amount of *S. aureus* with a SCVs-like phenotype, which resists the cellular degradation mechanism, leads to persistence of the bacteria [79]. SCVs have low antibiotic susceptibility and cause chronic or persistent infections, which complicates treatment efforts and warrants increased attention. By investigating the regulatory mechanisms underlying SCVs and their pathogenic effects on cells and tissues, as well as exploring effective detection methods and developing targeted anti-SCVs therapeutics, it is possible to achieve a reduction in the infection rate and incidence of SCVs-related infections.

## 4. The Ultimate Fate of *S. aureus*

After the vast majority of *S. aureus* are internalized into cells, they are either killed by lysosomes or escape the membrane-enclosed compartment, reaching a state of low replication. Some *S. aureus* will remain hidden in closed compartments with membrane structures to replicate, but it is unknown whether they reach the cytoplasm or are killed by acidic lysosomes [80]. At the same time, it was also found that a small amount of bacterial invasion also caused persistent infection of cells [80].

**Figure 5:**
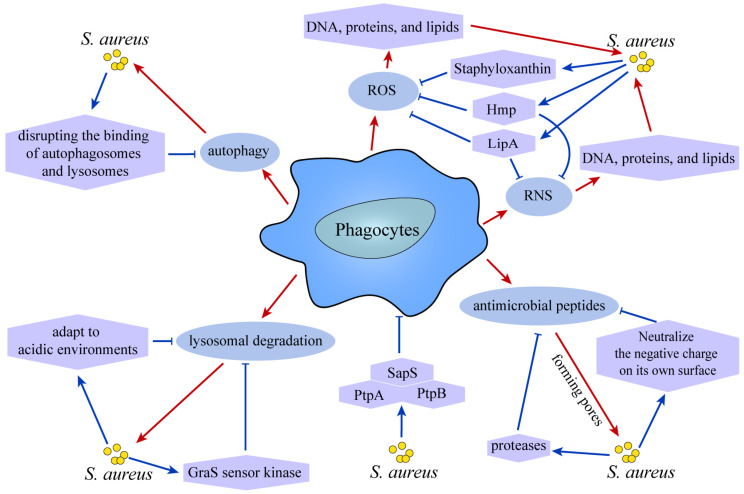
**Phagocytes strategies for killing *S. aureus* and the responses made by *S. aureus*.** Phagocytes production of ROS and RNS contributes to the clearance of *S. aureus*, the Staphyloxanthin of *S. aureus* protects *S. aureus* from ROS damage, and both LipA and Hmp produced by *S. aureus* inhibit phagocytes production of ROS and RNS. Phagocytes also secrete a number of cationic antimicrobial peptides that act to kill *S. aureus* by forming pores, while *S. aureus* produces molecules that bind to the antimicrobial peptides or secretes proteases that hydrolyze the antimicrobial peptides as well as neutralize the negative charge on the bacterial surface to evade the pore-forming activity of the antimicrobial peptides. Phagocytes also kill intracellular *S. aureus* by lysosomal degradation, with the involvement of cathepsins, and some *S. aureus* are able to adapt to the acidic environment and survive in the cell by means of the GraS sensor kinase. *S. aureus* also induces the onset of autophagy, which removes bacteria, but *S. aureus* take the appropriate measures to survive by disrupting the binding of autophagosomes to lysosomes. The protein tyrosine phosphatases PtpA, PtpB, and SapS secreted by *S. aureus* help *S. aureus* survive in phagocytes.

It has been shown that *S. aureus* will go through three stages in phagocytes: most of the bacteria will be degraded, and only a small percentage of bacteria will survive, the surviving bacteria will form clonal abscesses, and eventually the infection spreads in cells or tissues [81]. Phagocytes kill *S. aureus* through the following strategies: production of reactive oxygen species (ROS), reactive nitrogen species (RNS), as well as the secretion of specific antimicrobial peptides. Additionally, they utilize lysosomal degradation and autophagy as part of their antimicrobial arsenal. When *S. aureus* infects cells, it produces ROS, which is able to destroy some oxidizable parts of the bacteria’s DNA, proteins, and lipids, ultimately helping to get rid of the bacteria [82,83]. But at the same time, *S. aureus* has also made corresponding strategies to resist the destruction of ROS. Staphyloxanthin is a carotenoid pigment that acts as an antioxidant protecting *S. aureus* from ROS damage [84]. In immune cells, nitric oxide (NO) reacts with O^2−^ to produce nitrogen-based reactive substances and peroxynitros, and then nitric oxide synthase will produce RNS in the form of NO, which inhibits bacterial respiration and damages bacterial proteins and DNA, thereby exerting antimicrobial effects [85,86]. In NOS deficient mice, the incidence of *S. aureus* infection is notably increased. Lipoic acid (LipA) [86] and Flavonoid hemoglobin (Hmp) [87] produced by *S. aureus* both inhibit the production of ROS and RNS by macrophages to promote bacterial survival. Macrophages also secrete some cationic antimicrobial peptides, which play a bactericidal role by forming pores, and *S. aureus* will produce molecules bound to antimicrobial peptides or secrete proteases to hydrolyze antimicrobial peptides and neutralize the negative charge on the surface of the bacteria, evading the pore formation activity of antimicrobial peptides [88]. Low pH helps to kill intracellular bacteria and cathepsins are involved in the degradation of *S. aureus* [89]. Research implies that *S. aureus* strains USA300 and Newman possess the capability to acclimate to acidic environments, thereby gaining a growth advantage under such conditions [90]. Furthermore, it has been established that the GraS sensor kinase, a component of the GraXRS regulatory system, plays a pivotal role in the survival of *S. aureus* in acidic environments. Notably, this survival mechanism is not dependent on the α-toxin or PSMα, underscoring their limited significance in this context [91]. *S. aureus* can also induce the occurrence of autophagy, which is manifested by an increase in LC3 lipidation and an increase in P62 levels, indicating that it prevents autophagic flux, and after the use of autophagy inhibitors, the number of *S. aureus* in cells decreases, indicating that it survives by disrupting the binding of autophagosomes and lysosomes [92,93] (Figure 5). In the long-term infection of *S. aureus*, *S. aureus* will evade the killing of antibiotics, endolysosomes, and autophagy with different growth phenotypes, and after enhancing autophagy, *S. aureus* will have a second activation to escape the killing of the host cell, and after autophagy is inhibited, *S. aureus* will form a biofilm and reside in the cell, causing serious infection. Recently, studies have reported that bacteria secrete a number of phosphatases that play an important role in host-pathogen interactions. PtpA, a protein tyrosine phosphatase secreted by *S. aureus*, is one of the favorable factors in the survival of *S. aureus* in macrophages, where it undergoes binding to the host cell protein coronin-A (CorA), which is involved in *S. aureus* survival by influencing the spatial distribution of this actin-binding protein within the macrophage [94]. In a recent study, phosphorylation of PtpA was able to inhibit small ubiquitination modification (SUMOylation) of host cell proteins, which in turn promoted the survival of *S. aureus* in cells [95]. What exactly the role of PtpA in the cell is and its exact mechanism remains to be explored. Likewise, PtpB favors the survival of *S. aureus* [96,97]. The acid phosphatase SapS also contributes to the survival of *S. aureus* in macrophages, and when SapS was knocked down, the survival of *S. aureus* in cells was significantly reduced [98].

**Figure 6:**
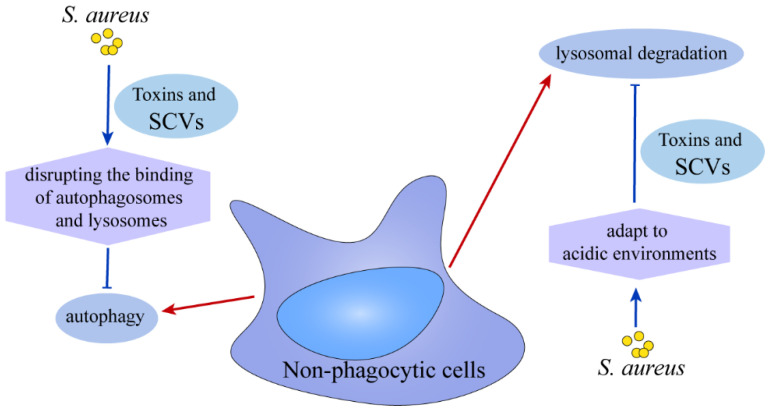
**Strategies used by non-phagocytic cells to kill *S. aureus* and the responses made by *S. aureus*.** Non-phagocytic cells eliminate intracellular *S. aureus* predominantly through lysosomal degradation or autophagy. In contrast, *S. aureus* predominantly employs the secretion of toxins or the formation of stable SCVs as mechanisms to evade lysosomal degradation and autophagy, thereby ensuring its survival.

In nonphagocytic cells, which lack antimicrobial properties like phagocytosis, *S. aureus* effectively inhibits the maturation of endosomes and the fusion of autophagic lysosomes, thereby maintaining its presence within the host cell [99,100]. Various strategies have been developed to regulate bacterial replication through the inhibition of autophagy. For instance, isozymes belonging to the serine/threonine kinase protein kinase C (PKC) family, which modulate Ca^2+^ signaling, play a significant role in autophagy induced by bacteria and are instrumental in hindering bacterial replication within host cells [101]. The application of the lysosomal alkalinizing agent hydroxychloroquine facilitated the elimination of *S. aureus* from osteoblasts [102]. In non-phagocytic cells, the prolonged existence of *S. aureus* in cells is dependent on the toxins secreted by it or the formation of stable SCVs [79]. *S. aureus* SCVs demonstrate the ability to persist over extended periods in the acidic conditions of lysosomes [103] (Figure 6).

## 5. Summary 

The investigation of *S. aureus* is increasingly gaining attention within the scientific community. This bacterium exhibits a remarkable capacity for global dissemination, which correlates strongly with its virulence factors. Nevertheless, the interplay between the host and these virulence factors is likely to be complex and multifaceted. It remains to be elucidated whether, at various stages of infection, the host is more significantly impacted by virulent or less virulent strains. Furthermore, the roles of individual toxins versus the synergistic effects of multiple toxins warrant careful examination. It is also pertinent to determine the specific stages of infection during which the bacteria may release fewer toxins while maintaining the ability to persist intracellularly for extended periods. Additionally, the mechanisms through which bacteria internalize host cells may involve one or multiple pathways. The factors that contribute to the persistence of bacterial infection, such as the diversity of strains, multiplicity of infection (MOI) values, and types of host cells, require further exploration. In summary, the study of the long-term infection and survival of *S. aureus* represents an ongoing challenge that necessitates continued investigation into the dynamics of various strains within different host cellular contexts. Presently, the examination of *S. aureus* internalization and survival within host cells is identified as a critical objective in addressing the infectivity and pathogenicity of this organism. This discussion aims to elucidate this intricate relationship and provide a foundation for future vaccine development and therapeutic strategies.

## 6. Complementary

The classical amino acid sequences of the above proteins were obtained using NCBI (https://www.ncbi.nlm.nih.gov/) or UniProt (http://uniprot.org) and studied using AlphaFold 3 (https://alphafoldserver.com/) docking and the accuracy of the predictions was judged by the pTM and ipTM scores. When the pTM score is above 0.5, it means that the predicted overall folded structure of the complex may be similar to the true structure. The ipTM measures the accuracy of the relative positions of the subunits within the predicted complex. ipTM values between 0.6 and 0.8 may or may not be correctly predicted. For protein interactions, ipTM + pTM ≥ 0.75 was considered a stronger interaction [104]. Here, it is shown that not all of the AlphaFold 3 predictions are correct, and it is still important to use the experimental results as the gold standard. Finally, PyMOL (TM) 3.0.4 is then used for visualization and analysis. All 3D models in this paper were constructed using AlphaFold 3 and, finally, visualized and analyzed using PyMOL. All figures presented in this paper were created using Adobe Illustrator 2020.

## Figures and Tables

**Table 1:** *S. aureus* effectors and their target host factors.

	Toxicogenic Proteins (Effectors)	Target Host Factors	Reference
Internalization	FnBp	α5β1	[9]
	Atl	Hsc70	[10]
	ClfB	AnxA2	[11]
	Lpls	Hsp90	[12]
	Tet38	CD36, TLR2	[13]
alpha-hemolysin (α-toxin)	α-toxin	ADAM10	[14,15]
		α5β1	[16]
		SYS1, ARFRP1, TSPAN14	[17]
		Tspan33, PDZD11, PLEKHA7, afadin	[18,19]
Phenol soluble modulins	PSM	TLR4	[20]
		FPR2	[21]
	PSMα3	TLR2, TLR4	[22]
Two-component leukotoxins	HlgAB	CXCR1, CXCR2, CCR2, CXCR4	[23]
	HlgCB	C5aR, C5L2	[23]
	LukED	CCR5	[15,24]
		CXCR1, CXCR2	[25]
	LukMF’	CCR1, CCR2, CCR5	[26]
	PVL	C5aR, CD88	[27]
	LukAB	CD11b	[28]

## Data Availability

All data generated or analyzed during this study are included in this published article.

## Supplementary Materials

The following supporting information can be downloaded at: https://www.mdpi.com/article/10.3390/ijms26020720/s1.
