# Supplementary material for: Strategies for Survival of Staphylococcus aureus in Host Cells
Source: Int J Mol Sci. 2025 Jan 16;26(2):720. doi: 10.3390/ijms26020720 (PMC11765632; doi:10.3390/ijms26020720)
Supplement: Supplementary file 1 [file ijms-26-00720-s001.zip › ijms-3406824-supplementary.pdf]

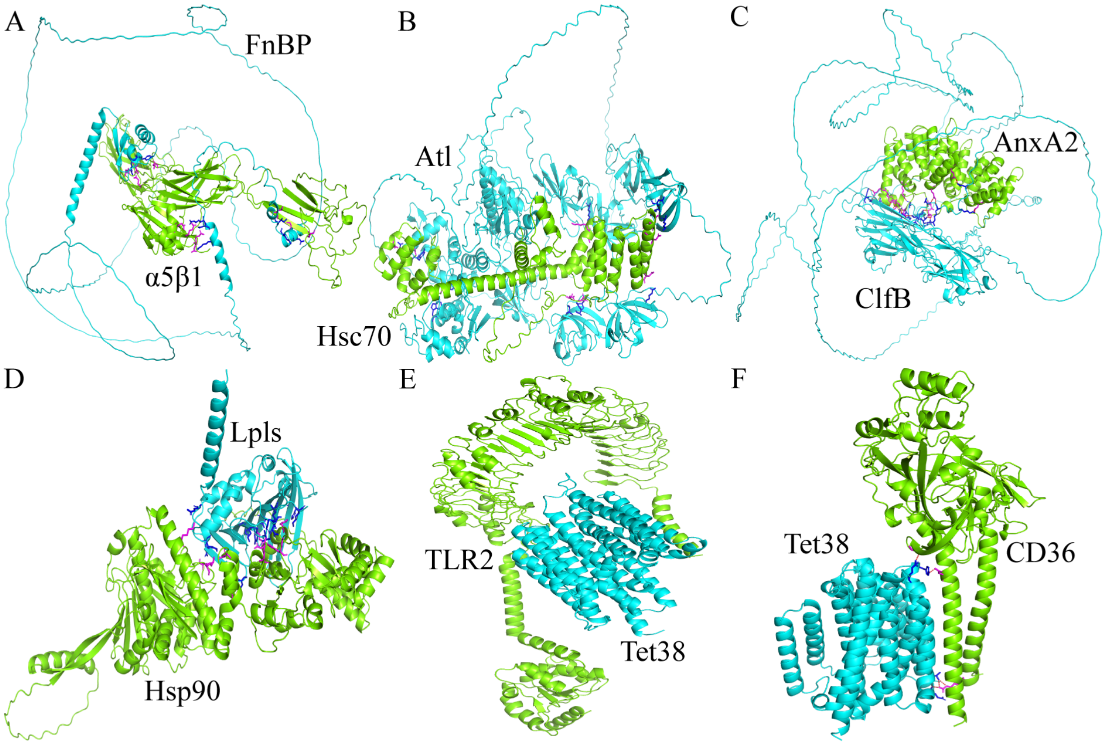

Figure S1. 3D structure of *S. aureus* adhesion factors bound to cell surface receptors upon internalization. A. 3D structure of FnBP bound to α5β1.B. 3D structure of Atl bound to Hsc70. C. 3D structure of ClfB bound to AnxA2. D. 3D structure of Lpls bound to Hsp90. E. 3D structure of Tet38 bound to TLR -2. F. 3D structure of Tet38 bound to CD36. Green is host surface receptor and cyan is the adhesion factor of *S. aureus*.

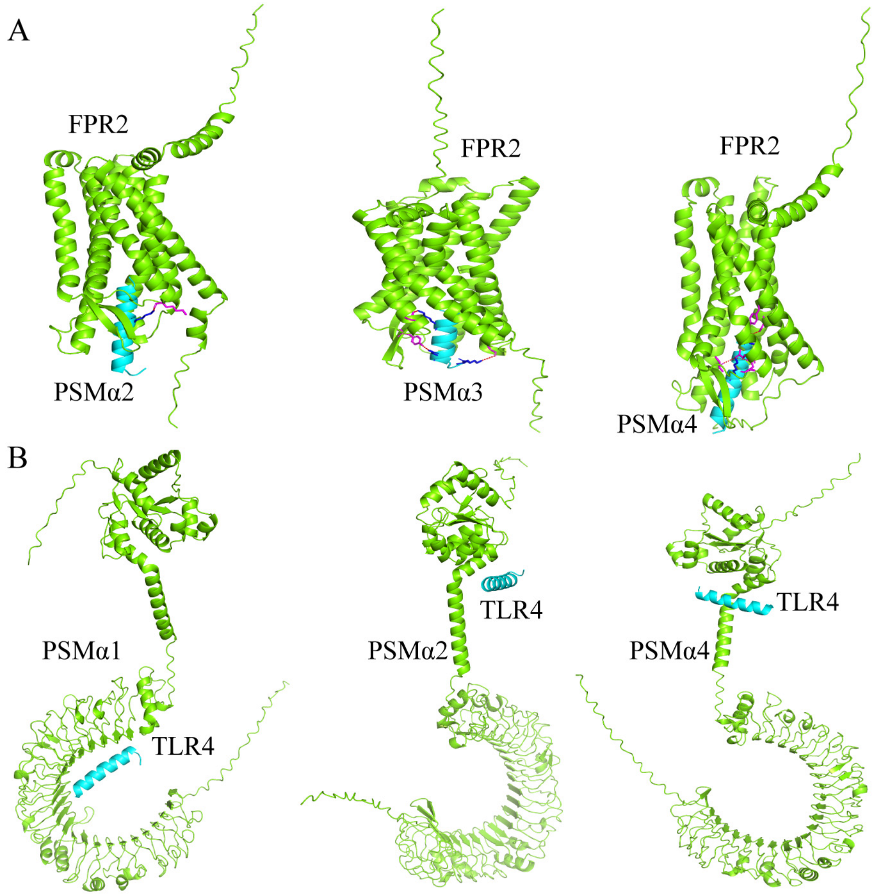

Figure S2. 3D structure of PSM binding to host proteins. A. 3D structure of PSM2-4 binding to FPR2. B. 3D structure of PSM1,2,4 binding to TLR4. Green is host protein, cyan is PSM.

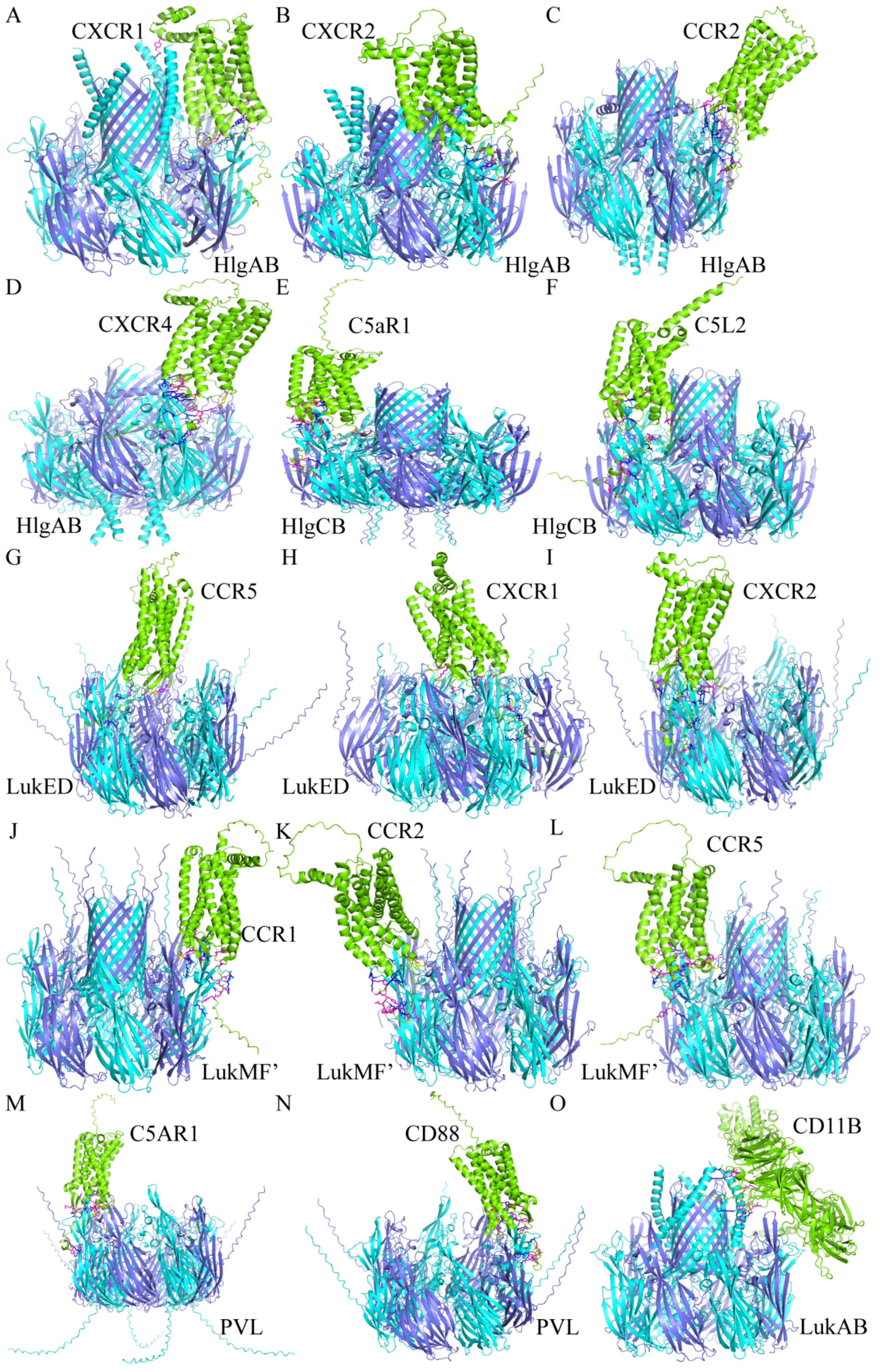

Figure S3. 3D structure of the two-component leukocidin with its bound surface receptor. A. 3D structure of HlgAB bound to CXCR1. B. 3D structure of HlgAB bound

to CXCR2. C. 3D structure of HlgAB bound to CCR2. D. 3D structure of HlgAB bound to CXCR4. E. 3D structure of HlgCB bound to C5aR. F. 3D structure of HlgCB bound to C5L2. G. 3D structure of LukED bound to CCR5. H. 3D structure of LukED bound to CXCR1. I. 3D structure of LukED bound to CXCR2. J. 3D structure of LukMF' bound to CCR1. K. 3D structure of LukMF' bound to CCR2. L. 3D structure of LukMF' bound to CCR5. M. 3D structure of PVL binding to C5aR. N. 3D structure of PVL binding to CD88. O. 3D structure of LukAB binding to CD11b. Green is the host surface receptor, cyan is the “S” subunit of leukocidin, and purple is the “F” subunit of leukocidin.

Table S1. Docking scores of effectors of *S. aureus* and their target host factors.

	Toxicogenic proteins (effectors)	Target host factors	iPTM	PTM	iPTM + PTM
Internalization	FnBp	α5β1	0.51	0.45	0.96
	Atl	Hsc70	0.18	0.31	0.49
	ClfB	AnxA2	0.7	0.5	1.2
	Lpls	Hsp90	0.3	0.55	0.85
	Tet38	TLR2	0.16	0.49	0.65
		CD36	0.19	0.51	0.7
alpha-hemolysin (α-toxin)	α-toxin	ADAM10	0.46	0.51	0.97
		α5β1	0.48	0.53	1.01
		ADAM10, SYS1, ARFRP1, TSPAN14	0.4	0.44	0.84
Phenol soluble modulins	PSMα1	FPR2	0.65	0.76	1.41
	PSMα2		0.57	0.76	1.33
	PSMα3		0.59	0.78	1.37
	PSMα4		0.56	0.77	1.33
	PSMα1	TLR4	0.41	0.67	1.08
	PSMα2		0.19	0.66	0.85
	PSMα3		0.23	0.68	0.91
	PSMα4		0.22	0.66	0.88
	PSMα3	TLR2	0.3	0.66	0.96
Two-component leukotoxins	HlgAB	CXCR1	0.59	0.62	1.21
		CXCR2	0.58	0.61	1.19
		CCR2	0.6	0.63	1.23
		CXCR4	0.61	0.64	1.25
	HlgCB	C5aR	0.75	0.76	1.51
		C5L2	0.75	0.77	1.52
	LukED	CCR5	0.26	0.33	0.59
		CXCR1	0.31	0.37	0.68
		CXCR2	0.27	0.34	0.61
	LukMF'	CCR1	0.54	0.57	1.11
		CCR2	0.54	0.57	1.11
		CCR5	0.53	0.57	1.1
	PVL	C5aR	0.32	0.38	0.7
		CD88	0.28	0.34	0.62
	LukAB	CD11b	0.47	0.51	0.98
